# Novel super-resolution capable mitochondrial probe, MitoRed AIE, enables assessment of real-time molecular mitochondrial dynamics

**DOI:** 10.1038/srep30855

**Published:** 2016-08-05

**Authors:** Camden Yeung-Wah Lo, Sijie Chen, Sarah Jayne Creed, Miaomiao Kang, Na Zhao, Ben Zhong Tang, Kirstin Diana Elgass

**Affiliations:** 1 Monash Micro Imaging, Monash University, Melbourne, Australia; 2 Hudson Institute of Medical Research, Clayton, Victoria, Australia; 3 School of Chemistry, University of Melbourne, Melbourne, Australia; 4 Division of Biomedical Engineering, Department of Chemistry, Hong Kong Branch of Chinese National Engineering Research Center for Tissue Restoration and Reconstruction, State Key Laboratory of Molecular Neuroscience and Institute of Molecular Functional Materials, The Hong Kong University of Science and Technology, Clear Water Bay, Kowloon, Hong Kong; 5 School of Chemistry & Chemical Engineering, Shaanxi Normal University, P.R. China

## Abstract

Mitochondria and mitochondrial dynamics play vital roles in health and disease. With the intricate nanometer-scale structure and rapid dynamics of mitochondria, super-resolution microscopy techniques possess great un-tapped potential to significantly contribute to understanding mitochondrial biology and kinetics. Here we present a novel mitochondrial probe (MitoRed AIE) suitable for live mitochondrial dynamics imaging and single particle tracking (SPT), together with a multi-dimensional data analysis approach to assess local mitochondrial (membrane) fluidity. The MitoRed AIE probe localizes primarily to mitochondrial membranes, with 95 ms fluorophore on-time delivering 106 photons/ms, characteristics which we exploit to demonstrate live cell 100 fps 3D time-lapse tracking of mitochondria. Combining our experimental and analytical approaches, we uncover mitochondrial dynamics at unprecedented time scales. This approach opens up a new regime into high spatio-temporal resolution dynamics in many areas of mitochondrial biology.

Mitochondrial dynamics are an area of intense interest in medical research^1^,^2^. Mitochondria are highly dynamic organelles that continuously undergo fusion and fission events, and are constantly redistributed in a cell to ensure regulated metabolite supply and cell viability^2^,^3^,^4^. A wide variety of severe human diseases including cancer^5^,^6^,^7^, cardiovascular diseases^8^,^9^,^10^, genetic and environmental metabolic diseases^11^,^12^,^13^,^14^ and neurodegenerative diseases^15^,^16^,^17^,^18^ have been linked to disordered mitochondrial dynamics, and mitochondrial repair/replacement therapy is of growing interest in modern medicine^19^, with the United Kingdom being the first country to pass legislation allowing for human mitochondrial transfer in IVF to treat mitochondrial diseases (http://www.legislation.gov.uk/ukdsi/2015/9780111125816/contents). Most of this research is based on high-resolution live cell fluorescence microscopy, as subcellular dynamics cannot be assessed in any other way. Emerging super-resolution optical techniques promise significant potential for mitochondrial dynamics research, as mitochondrial substructures such as cristae, are sized below the resolution limit of conventional confocal microscopy^20^,^21^. However, there has been very little application due to the lack of suitable dyes and the high laser excitation power needed for super-resolution imaging, which hampers applicability for live mitochondria imaging and requires highly specialized microscopes^22^,^23^,^24^. Here we present a novel mitochondria specific aggregation-induced emission (AIE) probe^25^, MitoRed AIE, suitable for live cell dSTORM (direct stochastic optical reconstruction microscopy) super-resolution imaging and assessment of mitochondrial dynamics and fluidity. MitoRed AIE (previously named TPE-Ph-In) is highly photostable and exhibits good biocompatibility. Our results demonstrate applicability of this probe for investigating mitochondrial dynamics in two different ways, single particle tracking (SPT) and mitochondrial super-resolution imaging. By combining both techniques we were able to visualize differences in mitochondrial membrane motility and thus assess local mitochondrial (membrane) fluidity, a characteristic assessment that has not been possible before with optical localization microscopy. We compared our results with that of a commercially available mitochondrial probe (MitoTracker Deep Red, Thermo Fisher Scientific) from the MitoTracker family of dyes, which are the most commonly used mitochondrial probes and the only ones that have been reported to be applicable for STORM super-resolution microscopy. We found our probe to be comparable or superior in all aspects tested. We expect this finding to have a revolutionary impact on mitochondrial dynamics research, as it catalyzes the full potential of super-resolution microscopy to this area.

## Results and Discussion

### Single Molecule Properties of MitoRed AIE

**Figure 1:**
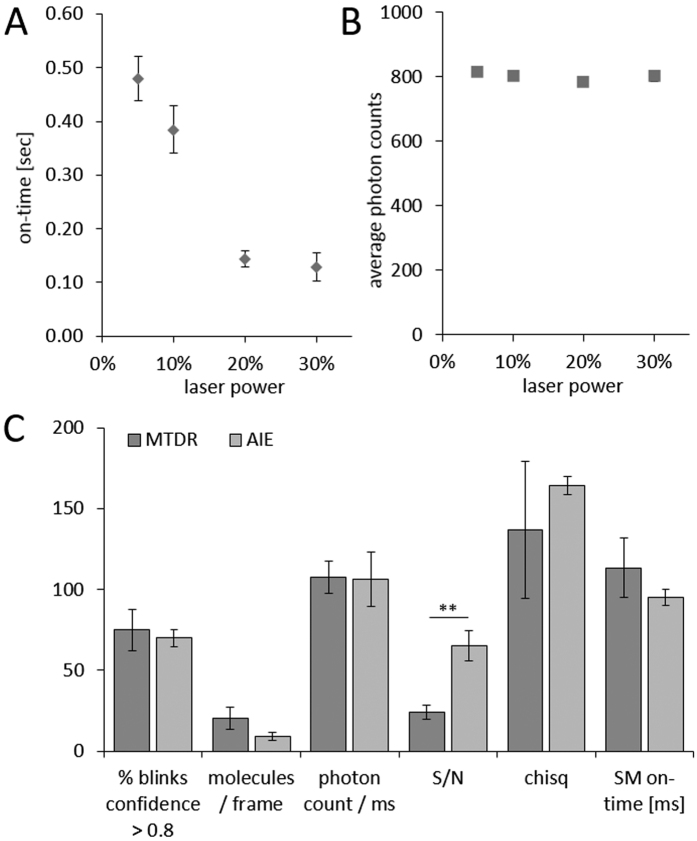
MitoRed AIE Single Molecule Statistics. (**A**) Average on-time of single MitoRed AIE dye molecules decreases significantly with increased excitation laser power while (**B**) average photon counts remain unaffected. (**C**) Single molecule blinking statistics of MitoRed AIE dye are similar or superior to MitoTracker Deep Red (MTDR). Signal-to-noise ratio (S/N) of single molecule blinks was 2.7 fold higher for our MitoRed AIE probe than for MTDR (p = 0.009, n = 8). **Indicates statistical significance with p < 0.01. As the y axis labeling differs for each of the experiments listed along x, the y axis labeling is included in the x axis description.

We first aimed to assess the photo-switching capabilities of the MitoRed AIE probe *in vitro* by imaging single MitoRed AIE molecules at various laser powers, determining the average brightness and on-time of single MitoRed AIE molecules. We found that the MitoRed AIE molecules readily go into dark states, with increasing excitation power significantly shortening the on-time (Fig. 1A), while the average number of photon emission counts per single molecule remained unaffected (Fig. 1B). The average on-time varied between 480 ms at 2 kW/cm^2^ laser power and 120 ms at 11 kW/cm^2^ laser power (measured at the back aperture of the objective).

From our single molecule experiments, we hypothesized that MitoRed AIE should be suitable for 3D dSTORM super-resolution in two different ways, for single particle tracking and assessment of mitochondrial membrane mobility, and for fast live dSTORM imaging of mitochondrial dynamics. We successfully applied both approaches to live cell mitochondrial dynamics.

### Localization of MitoRed AIE to Mitochondria and Mitochondrial Membranes

To verify MitoRed AIE’s specificity for mitochondria, we performed co-localization experiments with MitoRed AIE and various organelle markers (MitoTracker, LysoTracker and GFP-Sec61 as ER marker) to confirm mitochondrial localization (Supplementary Fig. S1). MitoRed AIE co-localized well with MitoTracker while no co-localization with either LysoTracker or GFP-Sec61 was observed.

MitoRed AIE is lipophilic and thus expected to have a higher affinity to lipids, especially negatively charged lipids, present in the membranes of mitochondria. The chemical structure of MitoRed AIE can be seen in Supplementary Fig. S2A. To test for the affinity of MitoRed AIE to lipids and specifically mitochondrial lipids, we performed an *in vitro* lipid affinity assay (see experimental procedures) using pure DOPC (dioleoyl phosphatidylcholine) lipids compared to DOPC with Cardiolipin. Cardiolipin constitutes about 20% of the total lipid composition of mitochondria and thus is a major component of the inner mitochondrial membrane^26^,^27^. We observed a two-fold increase of MitoRed AIE fluorescence intensity upon addition of Cardiolipin which supports our hypothesis of this dye’s significant mitochondrial membrane affinity (Supplementary Fig. S2B). This result is also consistent with the dSTORM images we obtained where membrane localization can most clearly be seen in images of fragmented mitochondria as those have much less cristae^28^ and therefore appear empty when imaged with a membrane probe.

### 3D Super-resolution Live Cell Imaging of Mitochondria with MitoRed AIE

**Figure 2:**
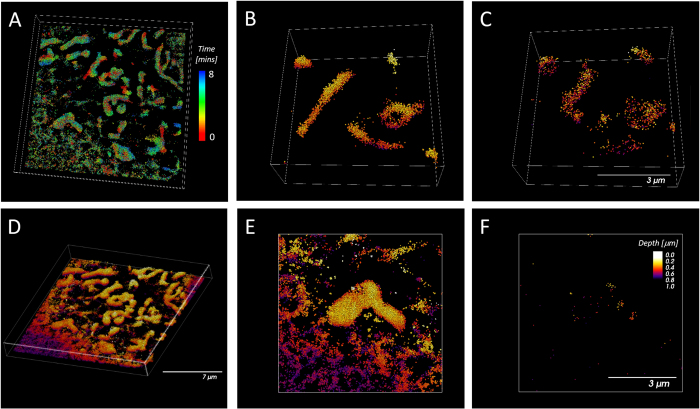
3D Live Cell Super-resolution Imaging of Mitochondria with MitoRed AIE Probe over 8 mins. (**A**) All detected localizations within 8 mins of imaging, color-coded for time. (**B**) Detected localizations in the first 10 sec of observation. (**C**) Detected localizations in the last 10 sec of observation. (**D**) 3D image of the same time series (all time points combined) color-coded for localization z-depth. (**E**) Magnified view of a single mitochondrion (all time points combined) color-coded for localization z-depth. (**F**) Magnified view of a single mitochondrion (localizations detected in 1 sec) showing intra-mitochondrial structure color-coded for localization z-depth.

In 2012 Shim *et al*. reported amongst other membrane probes, the use of MitoTracker Deep Red (MTDR) for high-speed dSTORM imaging^29^. Those experiments have been performed using a custom-built microscope, and to our knowledge have not been reproduced by other groups. Using commercial systems (Vutara SR350, Bruker and OMX Monet, GE Healthcare) with similar specifications, we could reproduce those results (MTDR: Supplementary Movie S1, MitoRed AIE: Supplementary Movie S2 and Fig. 2A–C) and compared the performance of our MitoRed AIE dye^25^ with MTDR to establish the viability of high-speed STORM imaging in live cells. MitoRed AIE was found to be similar or superior to MTDR in all assessed criteria (Fig. 1C and Supplementary Table S1). Average photon count of a single molecule was similar for both probes (MTDR = 108 cts/ms; MitoRed AIE = 106 cts/ms), however the signal-to-noise ratio (S/N) was about 3 times higher for MitoRed AIE due to lower background contribution (MTDR = 24; MitoRed AIE = 65). The fraction of single molecules that could be localized with confidence >0.8 (see Methods) was again similar for both probes (MTDR = 75%; MitoRed AIE = 70%). High photon counts and S/N ratio of single molecule blinks enabled reliable localization in z and thus 3D STORM imaging (Fig. 2D and Supplementary Movie S3) to observe 3-dimensional intra-mitochondrial dynamics (Fig. 2E,F and Supplementary Movies S4 (xy) and S5 (xz)) which to our knowledge has not been reported for any mitochondrial probes.

### Observation and Assessment of Mitochondrial Membrane Dynamics and Fluidity

**Figure 3:**
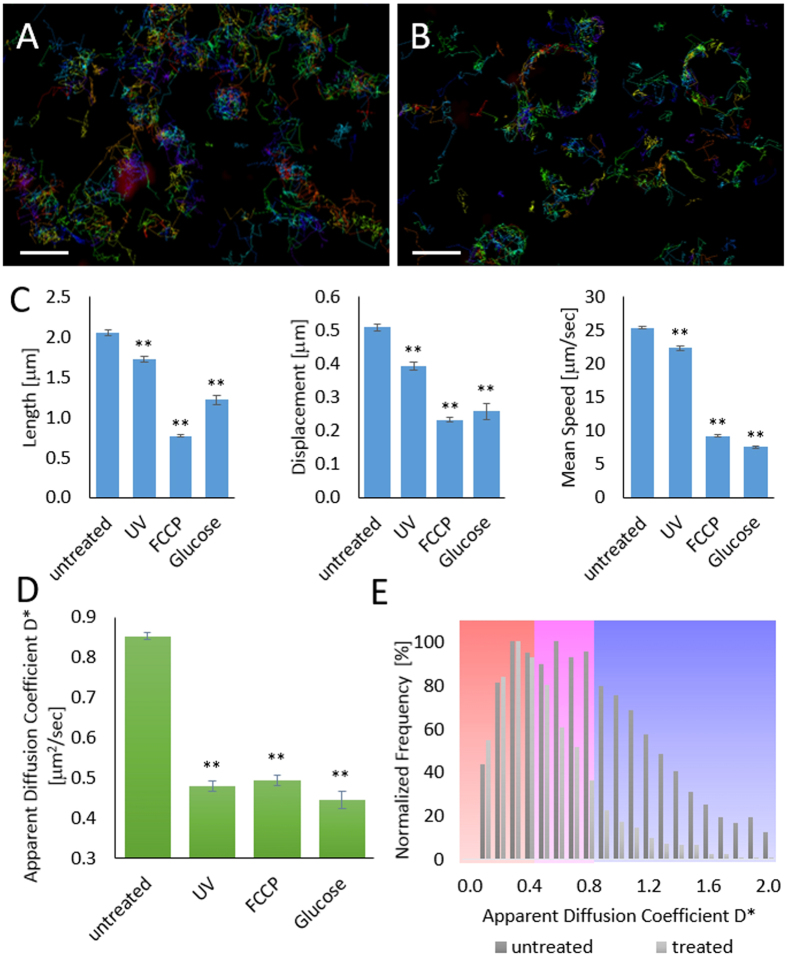
Comparison of Single Molecule Tracks in Healthy and Compromised Mitochondria. Single MitoRed AIE molecule tracks in (**A**) healthy and (**B**) compromised mitochondria. Scale bar is 1 μm. (**C**) All directional parameters (track displacement, length and mean speed) decrease in fragmented mitochondria (UV, FCCP, Glucose) compared to healthy mitochondria (untreated). **Indicates statistical significance with p < 0.01. (**D**) Apparent diffusion coefficient D* also decreases in impaired mitochondria (UV, FCCP, Glucose) compared to healthy mitochondria (untreated). **Indicates statistical significance with p < 0.01. (**E**) D* Histograms of healthy mitochondria show two peaks at 0.3 and 0.6 μm^2^/sec (dark grey bars), while D* histograms of treated mitochondria only show one peak at 0.3 μm^2^/sec (light grey bars). Background colors indicate color-coding according to slow (D* < 0.5, red), intermediate (D* > 0.5 and <0.8, magenta), or fast (D* > 0.8, blue) diffusion as indicated by background colors. Histograms are normalized to highest occurrence.

As MitoRed AIE is primarily membrane located, it should enable assessment of mitochondrial membrane dynamics as changes in membrane dynamics should affect the mobility of dye molecules. To test this hypothesis, we tracked single molecules in healthy and compromised mitochondria. Mitochondrial impairment was deliberately induced using various methods, firstly by exposing mitochondria to high 405 nm laser power (5 kW/cm^2^) until full fragmentation was observed (Fig. 3B and Supplementary Movie S6), secondly by treatment with FCCP (carbonylcyanide *p*-triflouromethoxyphenyl-hydrazone), a potent mitochondrial oxidative phosphorylation uncoupler that depolarizes mitochondria^30^, and thirdly by exposure to high glucose conditions (50 mM) to mimic hyperglycemia and induce mitochondrial ROS (reactive oxygen species) production^31^,^32^. For all three conditions we observed a significant decrease in mobility-dependent parameters such as track length (19–62%), displacement (20–60%) and mean speed (12–68%) in compromised compared to healthy mitochondria (Fig. 3C), indicating lower membrane mobility in compromised mitochondria. Notably, we performed the same experiment with healthy and fragmented mitochondria stained with MTDR and we did not observe any changes in MTDR track parameters which, as a non-membrane dye, supports our hypothesis. Supplementary Movie 7 shows the bulk of all AIE molecules in fast motion followed by single molecules in slow motion to highlight the high temporal resolution of the obtained data.

To further characterize the motility of MitoRed AIE single molecules in healthy and compromised mitochondria, we calculated motility curves^33^ and apparent diffusion coefficient D^*^^34^ for all detected SM tracks. We are aware that mitochondrial movement affects the measured diffusion coefficient. However, we rationalize that the individual track duration is short compared to the time scale of mitochondrial movement and thus D* would not significantly be affected by mitochondrial movement. To confirm this rationale we corrected a data set for mitochondrial movement (Supplementary Fig. S3) and did not observe significant changes in D* after correction. Average apparent diffusion coefficients D* also decreased significantly for all three methods of mitochondrial impairment compared to healthy mitochondria (Fig. 3D).

**Figure 4:**
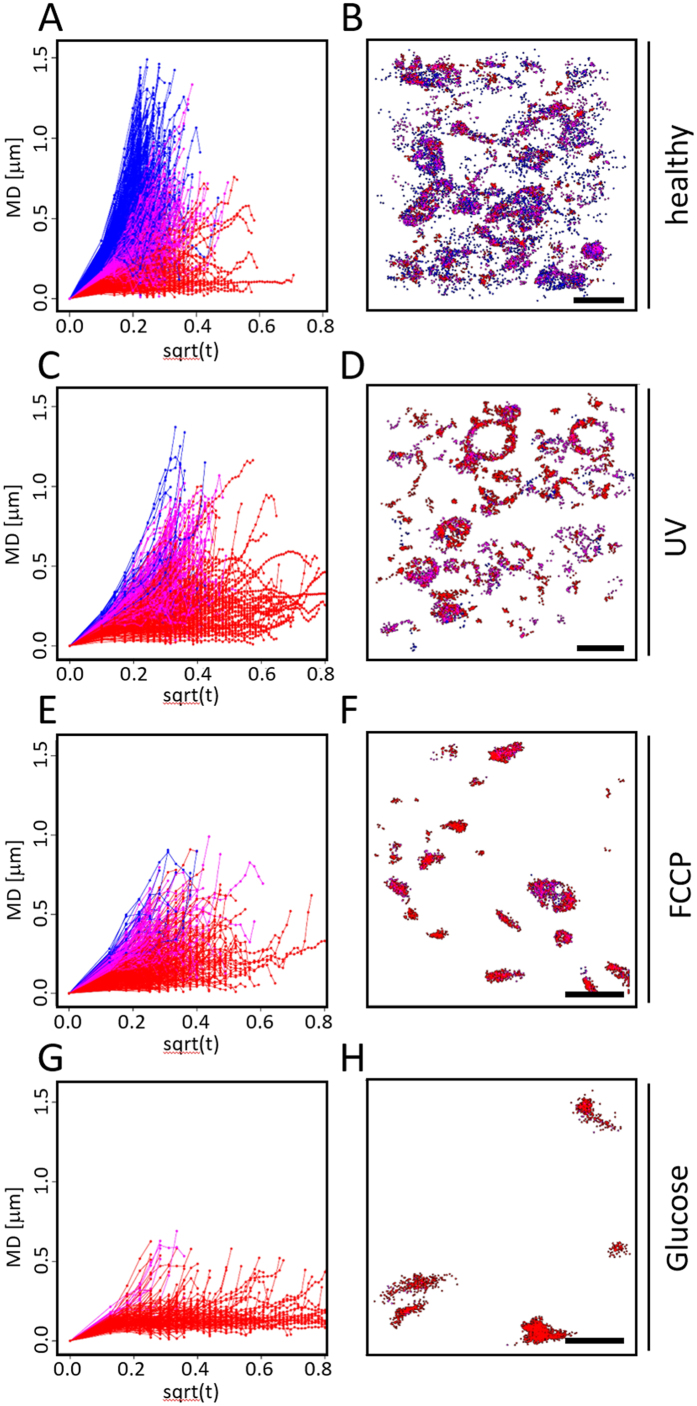
MitoRed AIE Single Molecule Dynamics in Healthy (**A,B**) and Compromised (**C**–**H**) Mitochondria. (**A,C,E,G**) Motility Curves of individual MitoRed AIE SM tracks, color-coded according to the respective apparent diffusion coefficient (red: D* < 0.5, magenta: D* > 0.5 and <0.8, blue: D* > 0.8) under different conditions as indicated on the right side of the panels. Most slowly diffusing molecules exhibit confined motion (red), while most fast diffusing molecules show directed movement (blue). (**B,D,F,H**) Localization of individual tracks, same color-coding as in (**A,C,E,G**). Regions of fast diffusion can be distinguished in healthy mitochondria (**B**) while SM tracks in fragmented mitochondria appear to have mostly confined character (**D,F,H**). Scale bars are 2 μm.

More detailed analysis revealed that D^*^ histograms of healthy mitochondria exhibit two peaks at 0.3 and 0.6 μm^2^/sec (Fig. 3E, dark grey bars), while D* histograms of treated mitochondria only show the peak at 0.3 μm^2^/sec (Fig. 3E, light grey bars). Based on this observation, we color-coded according to slow (D* < 0.5, red), intermediate (D* > 0.5 and < 0.8, magenta), or fast (D* > 0.8, blue) diffusion (as indicated by background colors in Fig. 3E). Most slowly diffusing molecules exhibited a confined character of movement, fast diffusing molecules showed directed movement Fig. 4A,C,E,G). By color-coding individual tracks according to their apparent diffusion coefficient in the reconstructed super-resolution image we were able visualize regions of various membrane mobility (Fig. 4B,D,F,H). As a reference, all tracks were also plotted rainbow-color-coded for track ID with each track having a different color to visualize individual SM tracks (Supplementary Fig. S4). With this approach of combining motility curve and apparent diffusion coefficient, we were able to further characterize the observed reduced membrane mobility in fragmented mitochondria. As can be seen in Fig. 4D,F,H upon fragmentation mitochondria are almost completely deprived of areas of directed, active movement indicating that these are linked with proper dynamics, distribution, and fusion of healthy mitochondria. To confirm our results we performed corresponding STED-RICS experiments^35^ (see Methods) and obtained similar values for AIE diffusion coefficients in fragmented and elongated mitochondria (Supplementary Fig. S5).

### 3D High-Speed Super-resolution Imaging of Mitochondrial Tubules

**Figure 5:**
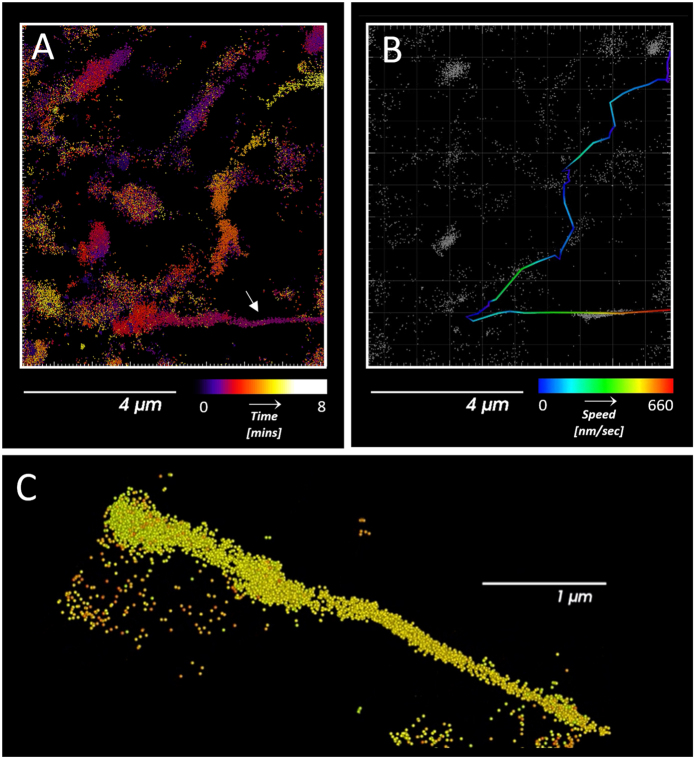
High-speed Super-resolution Imaging of Thin Mitochondrial Tubules. (**A**) Fast, thin mitochondrial tubule (white arrow) color-coded for time. (**B**) Same tubule tracked and color-coded for speed. Maximum speed of the tubule (red) was determined to be 660 nm/sec. (**C**) 3D magnified view of mitochondrial tubule shown in (**A**) at a different angle to allow easy assessment of its 3D sub-diffraction-limit dimensions. Its diameter was measured to be 100 nm in xy and 123 nm in z at position of highest speed.

For high-speed super-resolution imaging with the MitoRed AIE probe, we imaged mitochondrial dynamics for more than 8 mins (500 secs) without obvious light-induced fragmentation of the mitochondrial network (Fig. 2 and Supplementary Movie S2). Note that with MTDR we only managed to image for just over 3 mins (200 secs) without fragmentation of the mitochondrial network. Although Shim *et al*.^29^ report extended imaging periods of up to 10 mins as well, this was only possible under specific imaging conditions (when turning on illumination for one-fourth of the time with an excitation sequence of two frames of illumination followed by six dark frames^29^) while we imaged for about the same time period with standard continuous imaging conditions. The high single molecule photon emission counts of MitoRed AIE easily enable 3D imaging (biplane approach, Vutara SR350) without compromising imaging speed. Thus, we successfully imaged rapid dynamics of sub-diffraction limit sized mitochondrial tubules in 3D, as can be seen in Fig. 5 and Supplementary Movie S8. The diameter of the tubule was determined to be 100 nm in xy and 123 nm in z dimension (Supplementary Movie S9), the maximum speed of tubule movement was measured at 660 nm/sec.

## Conclusion

High-speed live cell super-resolution imaging is an emerging technique with outstanding potential to address questions in all areas of life sciences. The technique has yet to find wide applicability due to lack of suitable dyes and the necessity of highly specialized microscopes. Here we present a novel mitochondrial probe, MitoRed AIE, which is suitable for high-speed 3D super-resolution microscopy of live mitochondria, an extremely light-sensitive subcellular organelle, and therefore the most challenging target for this technique. Our characterization of MitoRed AIE within the STORM methodology context shows that it is an excellent super-resolution-capable mitochondria probe, surpassing that of MTDR. Furthermore, we utilize MitoRed AIE in practice, demonstrating live cell mitochondrial imaging, in 3D, at high temporal resolution, for extended continuous periods, all without compromising the viability of the observed cells. This is a significant leap in capability for studying live mitochondria in a high resolution spatio-temporal regime without compromise.

Our multi-dimensional data analysis approach of combining MitoRed AIE SPT and dSTORM imaging, specifically motility curves, apparent diffusion coefficient and track localization enables access to a new scale of mitochondrial dynamics different to any other type of mitochondrial dynamics reported so far. Within the average short on-time of single MitoRed AIE molecules mitochondria itself move very little, therefore changes in motility observed with this approach reflect local mitochondrial (membrane) fluidity rather than organelle dynamics making it a novel, unique approach in this field.

## Methods

### Cell Culture

COS-7 cells were cultured in complete DMEM with 10% FCS at 37 degrees and 5% CO_2_. For super-resolution microscopy COS cells were seeded in 8-well chamber slides on cover glass II from Sarstedt. Just before imaging, cells were stained with 5 μM MitoRed AIE in DMEM for 5 mins, staining media was then replaced with fresh phenol-red free DMEM for imaging and tiny amounts of glucose oxidase and catalase were added to the imaging media (0.4 μg/ml catalase and 5 μg/ml glucose oxidase) to serve as oxygen scavenger and promote returning of dye molecules from the dark state to the fluorescent state.

### *In Vitro* Lipid Affinity Assay

Lipids in chloroform were dried under a stream of nitrogen gas and then hydrated in PBS, pH 7.4, to a final lipid concentration of 2.2 mM. The lipids were incubated at 37 °C for 30 min, followed by bath sonication for 1 h. The lipids were then diluted to 22 μM with PBS and 0.1% MitoRed AIE (5 mM in DMSO stock solution resulting in a final concentration of 5 μM) was added to the lipids. The fluorescence spectra of the lipid-probe mixture were recorded on a Perkin-Elmer LS 55 spectrofluorometer.

### Microscopy

dSTORM super-resolution imaging was performed either on a Vutara SR350 system (Bruker) or on an OMX Monet system (GE Healthcare) using a 561 nm (568 nm on OMX system) or a 639 nm laser for excitation (MitoRed AIE and MTDR probe respectively) and a 405 nm laser for dye re-activation. A 60x TIRF, 1.49 NA oil objective was used for imaging. Camera exposure times was 5 ms or 10 ms for live cell imaging and SPT.

#### Single Molecule Imaging

MitoRed AIE stock solution (5 mM in DMSO, described in Zhao *et al*.^25^) was highly diluted to a final concentration of 5 nM. 5 μl of the diluted solution was deposited into a single well of an 8-well chamber slide on cover glass II from Sarstedt and imaged with various laser powers (2–11 kW/cm^2^) in TIRF mode. 5000 frames at an exposure time of 20 ms were recorded per field-of-view. For each laser power a new field-of-view was chosen.

#### Single Particle Tracking and High-Speed STORM Imaging

For single particle tracking all dyes were first pumped into the dark state at high laser powers (11 kW/cm^2^), then laser power was reduced to 2–4 kW/cm^2^, individual dye molecules were reactivated with low amounts of 405 nm laser light (10 W/cm^2^). 405 nm laser power was increased in 10 W/cm^2^ intervals over the imaging time to keep the number of detected blinks at approximately the same level. Single molecules were tracked for at least 50 ms (5 frames) and up to 1.8 s (180) frames.

STED-RICS super-resolution microscopy was performed on an Abberior easy3D STED system with 561 nm excitation laser, 775 nm STED laser and 100 × 1.4 NA oil objective. A time series of 30 STED-RICS images was recorded at 5% STED laser power, 10 nm pixel size and 10 μs pixel dwell time. Diffusion coefficients were calculated from regions of interest in the STED-RICS image series using previously published Matlab software^36^,^37^ (http://www.mathworks.com/matlabcentral/fileexchange/48703-raster-image-correlation-spectroscopy). Regions of interest were larger than 1 μm^2^.

### Image Reconstruction, Processing and Data Analysis

3D dSTORM superresolution data was reconstructed and processed using Vutara SRX software. Obtained single molecule localizations were denoised (threshold 0.3) and filtered for high confidence (>0.8) for visualization and further analysis. Single molecule data were analysed using Imaris software (Bitplane AG). Motility curves were calculated as described in Sumen *et al*.^33^, the apparent diffusion coefficient was calculated according to D* = MSD/(4Δt) (MSD: mean squared displacement)^34^. We note that we determine an apparent diffusion coefficient, not an accurate organelle component diffusion coefficient. Factors such as the topology of mitochondria, localization error and mitochondrial movement are part of the convolved observation.

## Additional Information

**How to cite this article**: Lo, C. Y.-W. *et al*. Novel super-resolution capable mitochondrial probe, MitoRed AIE, enables assessment of real-time molecular mitochondrial dynamics. *Sci. Rep.* **6**, 30855; doi: 10.1038/srep30855 (2016).

## Supplementary Material

Supplementary Information

Supplementary Movie S1

Supplementary Movie S2

Supplementary Movie S3

Supplementary Movie S4

Supplementary Movie S5

Supplementary Movie S6

Supplementary Movie S7

Supplementary Movie S8

Supplementary Movie S9
