# Supplementary material for: Novel super-resolution capable mitochondrial probe, MitoRed AIE, enables assessment of real-time molecular mitochondrial dynamics
Source: Sci Rep. 2016 Aug 5;6:30855. doi: 10.1038/srep30855 (PMC4974624; doi:10.1038/srep30855)
Supplement: Supplementary Information [file srep30855-s1.pdf]

Supplementary Material to:

Novel super-resolution capable mitochondrial probe, MitoRed AIE, enables assessment of real-time molecular mitochondrial dynamics

Camden Yeung-Wah Lo[1,2], Sijie Chen[3,4,5], Sarah Jayne Creed[1,2], Miaomiao Kang[4], Na Zhao[6], Ben Zhong Tang*[4], Kirstin Diana Elgass*[1,2]

	**MTDR Mean**	**MTDR StdDev**	**MTDR S.E.M.**	**AIE Mean**	**AIE StdDev**	**AIE S.E.M.**	**t-test p-value**
% single molecules detected with confidence > 0.8	75	22	13	70	12	5	0.97
detected molecules [1/10ms]	20	12	7	18	10	5	0.93
photon counts [1/ms]	108	17	10	106	38	17	0.95
**S/N ratio**	**24**	**8**	**4**	**65**	**21**	**9**	**0.01**
chisq	137	73	42	164	13	6	0.58
single molecule on-time [ms]	113	32	19	95	10	5	0.35

Supplementary Table S1: MitoRed AIE vs MTDR Single Molecule Statistics. Comparison of blinking statistics in live cells for MitoRed AIE and MTDR. Both probes are similar with MitoRed AIE being vastly superior in signal-to-noise ratio of single blinks (highlighted in bold). P-values of a two-tailed t-test are given in the last column.

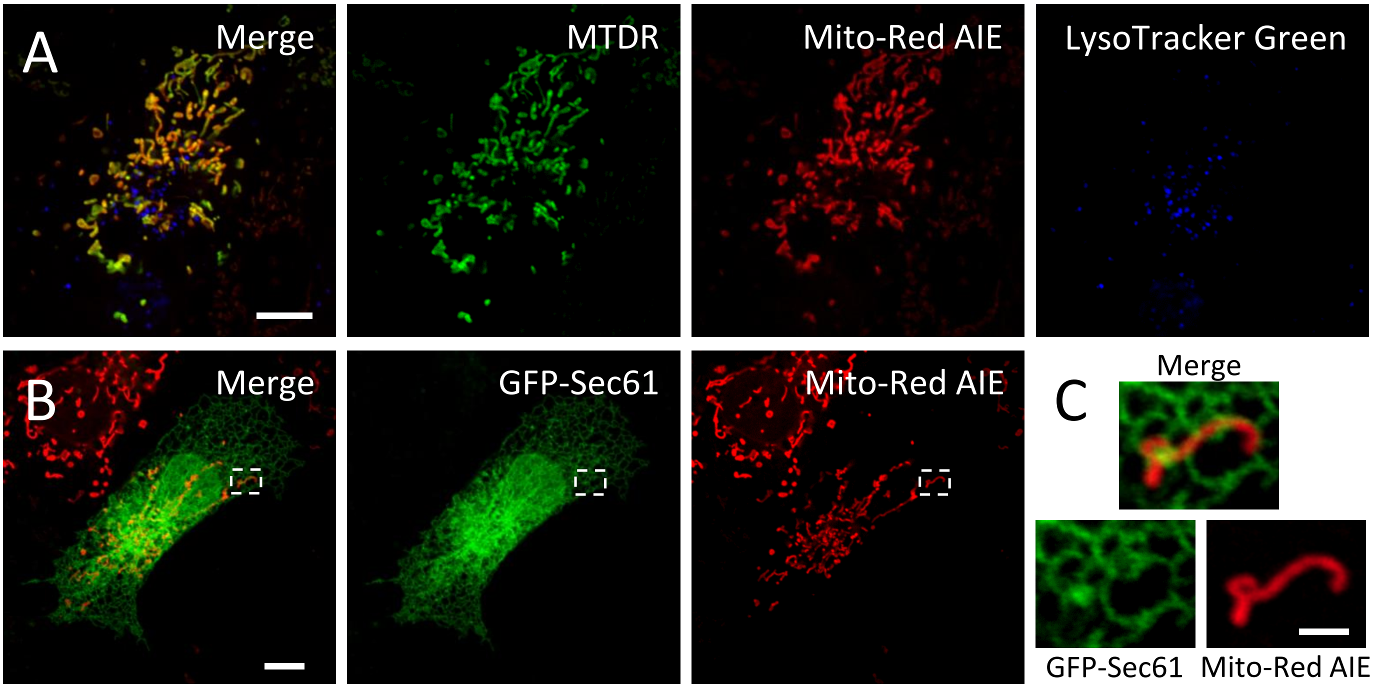

Supplementary Figure S1: Co-localization of MitoRed AIE with Organelle Markers. (A) Confocal image of Mito-Red AIE (red) with MitoTracker Deep Red (green) and LysoTracker Green (blue). Scale bar is 10μm. (B) Confocal image of MitoRed AIE (red) with GFP-Sec61 as ER marker (green). Scale bar is 10μm. (C) Magnified view of MitoRed AIE and ER as indicated with the white box in (B). Scale bar is 2μm.

A

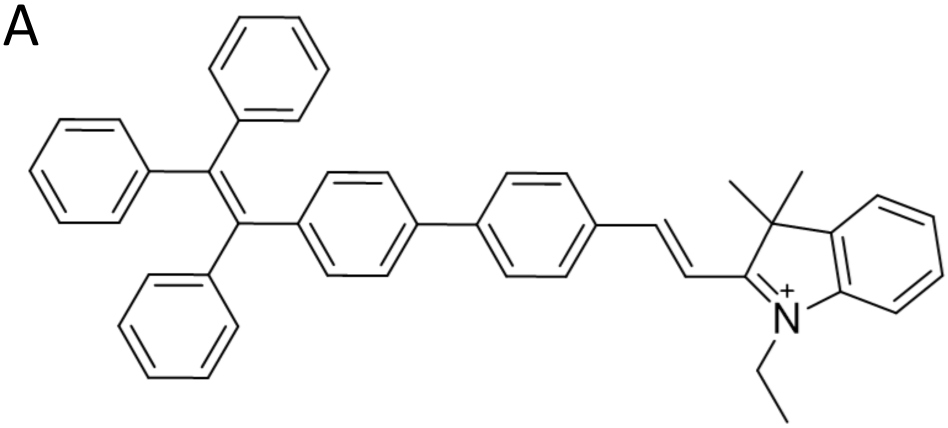

B

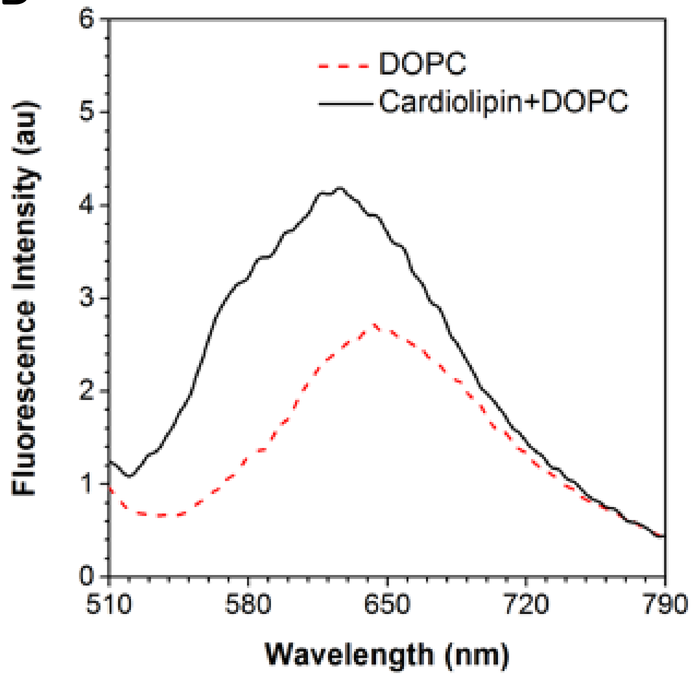

Supplementary Figure S2: Chemical structure and *In vitro* lipid affinity assay of MitoRed AIE. (A) MitoRed AIE is a lipophilic probe and is expected to have a higher chance to interact with and target lipids, especially the negatively charged lipids, in the membranes of the mitochondria. (B) Emission spectra of MitoRed AIE in the presence of DOPC or Cardiolipin/DOPC (1:1) mixture. MitoRed AIE shows a two-fold increase in fluorescence with DOPC and Cardiolipin, which is a major component of the mitochondrial inner membrane, compared to DOPC alone.

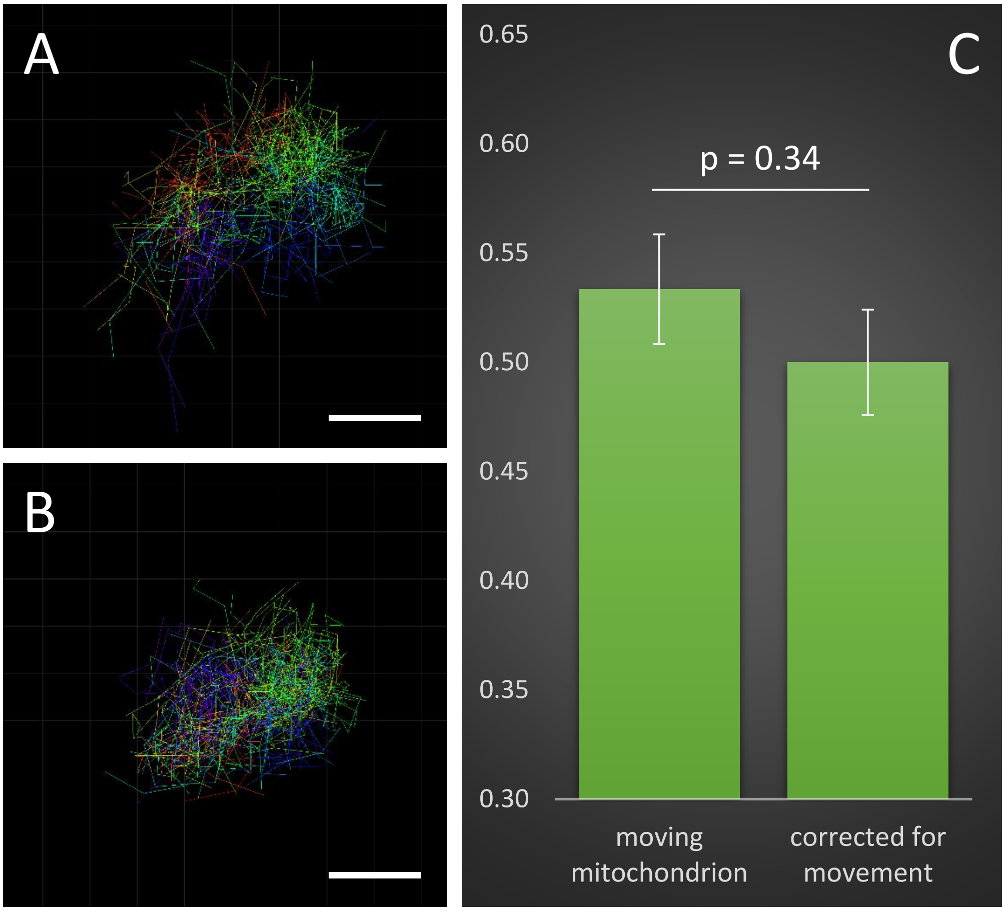

Supplementary Figure S3: Mitochondrial Movement Correction of Diffusion Coefficients. (A) Single molecule tracks detected in a moving mitochondrion color-coded for time. (B) Single molecule tracks detected in a mitochondrion corrected for mitochondrial movement and color-coded for time. Scale bars are 0.2 μm. (C) Apparent diffusion coefficients D* of single molecules show no statistically relevant changes after correction.

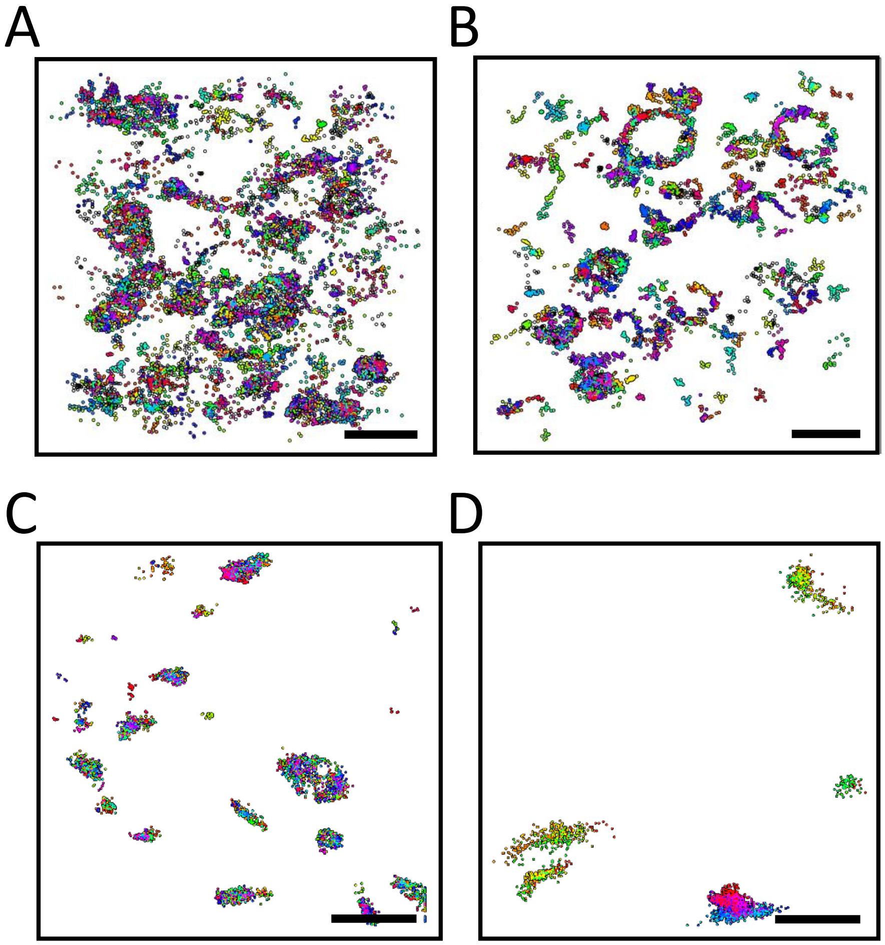

Supplementary Figure S4: Single MitoRed AIE Molecule Tracks rainbow-color-coded for track ID with each track having a different color to visualize individual SM tracks.

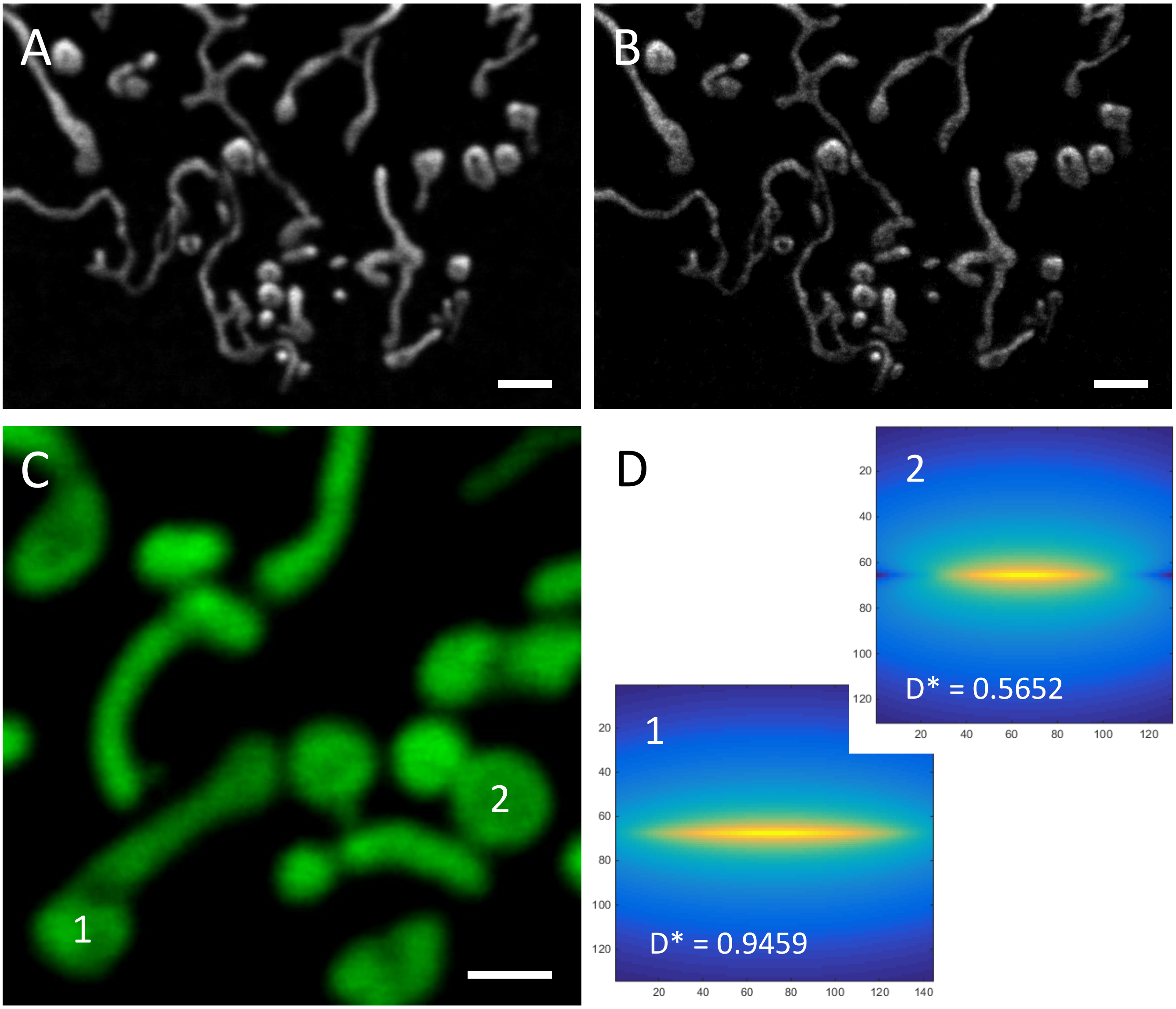

Supplementary Figure S5: MitoRed AIE STED and STED-RICS. (A) Confocal image of mitochondria stained with MitoRed AIE (B) Corresponding STED image. Scale bar is 2μm. (C) First frame of 30 frames of a recorded STED-RICS time series. One elongated and one fragmented mitochondrion was chosen and analysed with RICS (D) STED-RICS correlation maps and calculated diffusion coefficients for regions 1 and 2.

Supplementary Movie S1: Time Series of 3D Live Cell Super-resolution Imaging of Mitochondria with MTDR Probe over 200 secs. Movie starts off showing all detected molecules color-coded for time, then changes to depth-color-coding, followed by the corresponding time series to visualize mitochondrial dynamics.

Supplementary Movie S2: Time Series of 3D Live Cell Super-resolution Imaging of Mitochondria with MitoRed AIE Probe over 500 secs. Movie is color-coded for depth.

Supplementary Movie S3: 3D View of Time Series (all time points) shown in Supplementary Movie S2. Movie is color-coded for time, time is given in frames with 10ms/frame.

Supplementary Movie S4: 3D Imaging of Intra-Mitochondrial Dynamics with MitoRed AIE (xy plane). Movie starts off showing all detected molecules color-coded for depth, then changes to the corresponding time series to visualize intra-mitochondrial dynamics. This movie shows a magnified view of a single mitochondrion in Supplementary Movie S2 with a different time resolution.

Supplementary Movie S5: 3D Imaging of Intra-Mitochondrial Dynamics with MitoAIE Red (xz plane). Movie starts off showing all detected molecules over time, then changes to the corresponding time series to visualize intra-mitochondrial dynamics in xz.

Supplementary Movie S6: Light-induced Mitochondrial Fragmentation. Movie starts off showing all detected molecules color-coded for time, then changes to the corresponding time series to visualize mitochondrial fragmentation. Time is given in frames with 10ms/frame.

Supplementary Movie S7: 3D Imaging of MitoRed AIE Single Molecules. Movie starts off showing all detected molecules color-coded for depth, then changes to showing movement of individual mitochondria, then changes again to show movement of individual molecules. The second part of the movie (mitochondrial movement) plays 30 times faster than real-time, while the third part (single molecule movement) plays 6 times slower than real-time.

Supplementary Movie S8: High-speed Super-resolution Imaging of Thin Mitochondrial Tubule. Movie starts off showing all detected molecules color-coded for time, then changes to the corresponding time series to visualize mitochondrial fragmentation. Time is given in frames with 5ms/frame.

Supplementary Movie S9: 3D View of Single Molecules of MitoRed AIE which trace out the Thin Mitochondrial Tubule shown in Supplementary Movie S5. 3D magnified view of mitochondrial tubule at different angles allows easy assessment of its 3D sub-diffraction-limit dimensions. Its diameter was measured to be 100 nm in xy and 123 nm in z at position of highest speed.
